# Does the use of epicutaneous vacuum-assisted closure after revision surgery on the spine reduce further wound revision surgery?

**DOI:** 10.1007/s00264-023-05695-z

**Published:** 2023-01-17

**Authors:** Stefan Gläsel, Jan-Sven Jarvers, Philipp Pieroh, Christoph-Eckhard Heyde, Ulrich J. Spiegl

**Affiliations:** 1 Department of Spine Surgery and Neurotraumatology, Sana Klinikum Borna, Borna, Germany; 2 Department of Orthopaedics, Trauma Surgery and Plastic Surgery, University of Leipzig, Liebigstr. 20, 04103 Leipzig, Germany

**Keywords:** Epicutaneous vacuum therapy, Surgical site infection, Wound revision surgery

## Abstract

**Purpose:**

This study aimed to investigate the effect of epicutaneous vacuum therapy on the rate of unplanned spinal wound revisions compared with conventional wound dressing.

**Methods:**

This retrospective study included patients who underwent unplanned revision spine surgery after primary aseptic spine surgery who were treated at a level I spine centre between December 2011 and December 2019. Patients with revision surgery who required a further unplanned revision surgery during the inpatient stay were considered a treatment failure. The epicutaneous vacuum-assisted closure (Epi-VAC) therapy was the standard treatment method beginning in 2017 (the epi-VAC group). Before, conventional wound dressing was used (the control group (CG)). In addition, a one-to-one matched-pair comparison analysis was performed between both groups.

**Results:**

Of 218 patients, 48 were in the epi-VAC group. The mean age was 65.1 years (epi-VAC 68.2 to CG 64.3 years (*P* = 0.085)), and the mean body mass index (BMI) was 28.2 kg/m^2^ (epi-VAC 29.4 to CG 27.9 kg/m^2^ (*P* = 0.16)). No significant differences in the treatment failure rate could be detected between the two groups (epi-VAC 25% to CG 22.4% (*P* = 0.7)). There was also no significant difference for the matched-pair analysis (epi-VAC 26.1% to CG 15.2% (*P* = 0.3)). An elevated CRP level (C-reactive protein) immediately before the first wound revision was a significant risk factor for further revision surgery (treatment failure: 135.2 ± 128.6; no treatment failure: 79.7 ± 86.1 mg/l (*P* < 0.05)).

**Conclusion:**

Concerning repeat unplanned wound revision after spinal revision surgery, we cannot demonstrate an advantage of the epicutaneous vacuum therapy over conventional wound dressing.

## Introduction

Post-operative surgical site infections (SSI) are the third most common type of nosocomial infection in acute care hospitals [1]. They cause an increase in both morbidity and mortality as well as a prolonged hospital stay and lead to higher treatment costs [2].

Quantity, type, and pathogenicity of the (primarily bacterial) pathogen, existing infection-promoting circumstances of the patient, and surgical technical conditions influence the occurrence of post-operative wound infections [3]. According to data from the “German National Reference Center for Surveillance of Nosocomial Infections,” 103 (0.5%) wound infections occurred in 22,838 lumbar disc surgeries between January 2017 and December 2019. A total of 382 (3%) wound infections occurred during the same period in 12,530 spondylosyndesis procedures [1].

Superficial (epifascial) wound infections can be treated conservatively in most cases. However, if deep (subfascial) wound infections occur, wound revision with aggressive debridement, irrigation, and drainage is necessary [4]. The epicutaneous vacuum-assisted closure (epi-VAC) therapy, applying a vacuum dressing to a closed surgical wound, is discussed as a treatment modality that might reduce SSI and the rate of re-operations. The scientific interest in this type of wound dressing is steadily increasing [5]. In contrast, there are only a few studies on the epi-VAC therapy in spine surgery. These showed divergent results concerning the prevention of wound dehiscences and infections using the posterior approach [6–8]. The rate of re-operations differed statistically and non-significantly [6, 7].

We are unaware of any currently available literature studies investigating whether the epi-VAC therapy affects the rate of re-operations after revision spine surgery. Based on the experience of our clinical work, we suspected that re-operations could be reduced by the epi-VAC treatment. To verify this assumption was the aim of the present work.

## Methods

This retrospective study was conducted in a level I spine center between December 2011 and December 2019.

Patients who were 18 years and older and underwent primary aseptic spine surgery and revision surgery because of a wound-healing disorder (wound infection/dehiscence/seroma) during a single inpatient stay were included.

Exclusion criteria were initial surgery due to infections, ventral approaches, and procedures involving instrumentation of the os sacrum. Furthermore, we excluded patients who received revision surgery due to material-related complications.

**Fig. 1:**
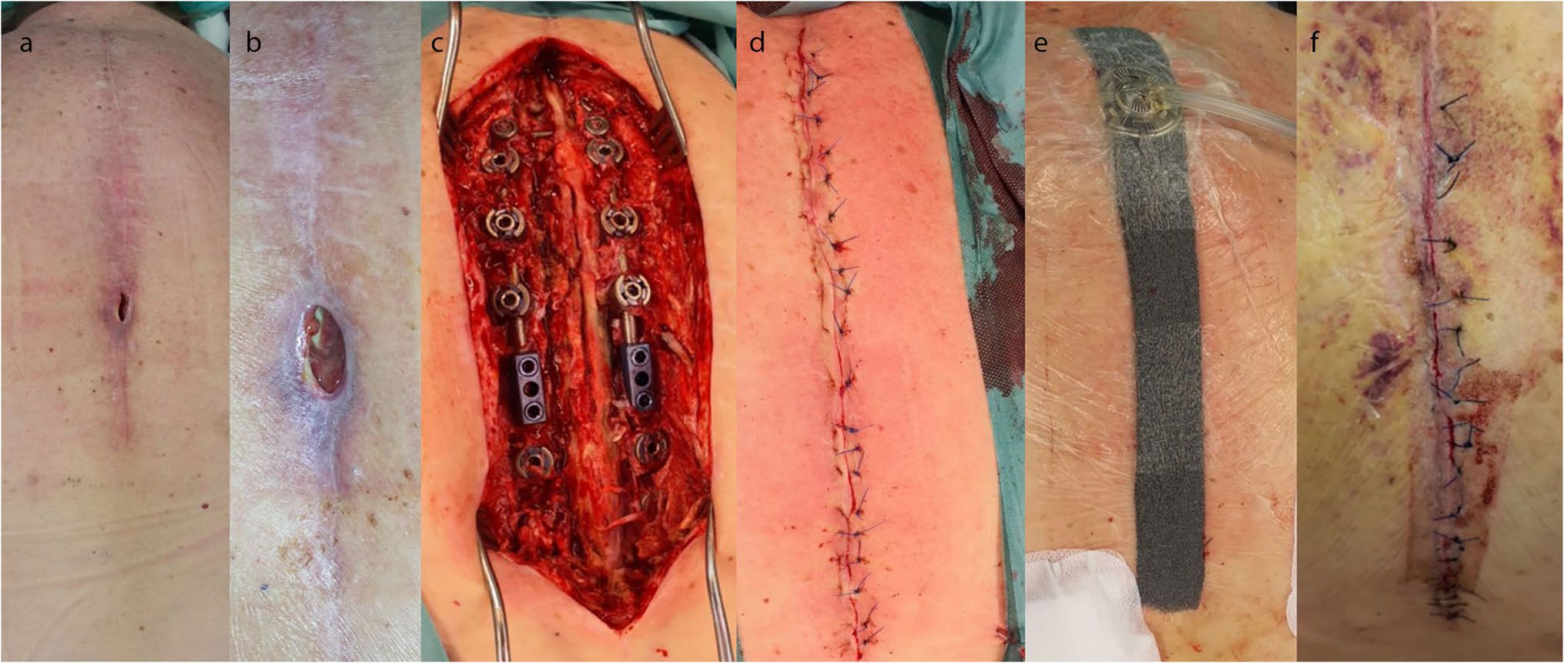
A 76-year-old patient with an adjacent fracture of Th10 after suffering a Th12 fracture initially. Sixteen days after posterior stabilization, a fistula at the posterior approach was visible (**a**, **b**). Surgical revision was performed including radical wound debridement, jet lavage, and local and systemic antibiotic therapy (**c**). After secure fascia closure and wound closure by sutures (**d**), the epicutaneous vacuum therapy was applied for 7 days (**e**). The wound situation was dry after the removement of the vacuum therapy with some areas of skin irritation at the borders of the vacuum therapy (**f**).

We included 218 patients and divided them into two groups. Between 2011 and 2016, patients received a fleece surgical wound dressing as standard (the control group (CG)). Since 2017, we have applied an epicutaneous vacuum dressing following revision surgery in all patients without changing the further operative and peri-operative treatment (the epi-VAC group shown in Fig. 1).

Further therapeutic measures in post-operative wound-healing disorders were identical (radical wound debridement, jet lavage, local and systemic antibiotic therapy, and secure fascia closure). Intra-operatively, we swabbed the wound superficially and in a subfascial manner.

We collected patient-related data and comorbidities already assessed as relevant in the previous studies [9–14]: the American Society of Anesthesiologists (ASA) classification, nicotine abuse, arterial hypertension, coronary artery disease (CAD), peripheral arterial disease (PAVD), chronic obstructive pulmonary disease (COPD), osteoporosis, active cancer, radiation/chemotherapy, systemic cortisone therapy, and anticoagulation.

In addition, we examined hospital-related parameters: extent of primary surgery, number of addressed vertebral bodies, duration of initial and revision surgery, the time between first and second wound revisions, number of revisions, local antibiotic therapy, and the mortality rate.

Before the first revision surgery, we immediately collected the expression of laboratory values (haemoglobin, leukocytes, albumin, and C-reactive protein).

To reduce patient group bias, we performed a one-to-one matched-pair comparison analysis. We identified 46 patients, each of whom could be assigned a matching partner considering ten characteristics, and implemented a scoring system. We evaluated the ASA score, age, and body mass index to be particularly relevant. Additionally, we gave additional individual scores in descending rank order for concordance in a pressure ulcer, length of ICU stay after first wound revision, length of hospital stay, germ, diabetes, renal insufficiency, and sex.

Initially, we compared each epi-VAC patient with each patient in the control group. The sum of the individual scores for the ten characteristics under consideration yielded a personal similarity score for each possible pairing. The aim was to identify pairings for which the sum of all scores was maximal (as shown in Table 1). This maximum total score was decisive for the final matching, so a similar match was occasionally omitted to obtain a higher total score (global-optimal matching). We did not find matching partners for two epi-VAC patients due to different ASA scores under the established limits.

**Table 1:**
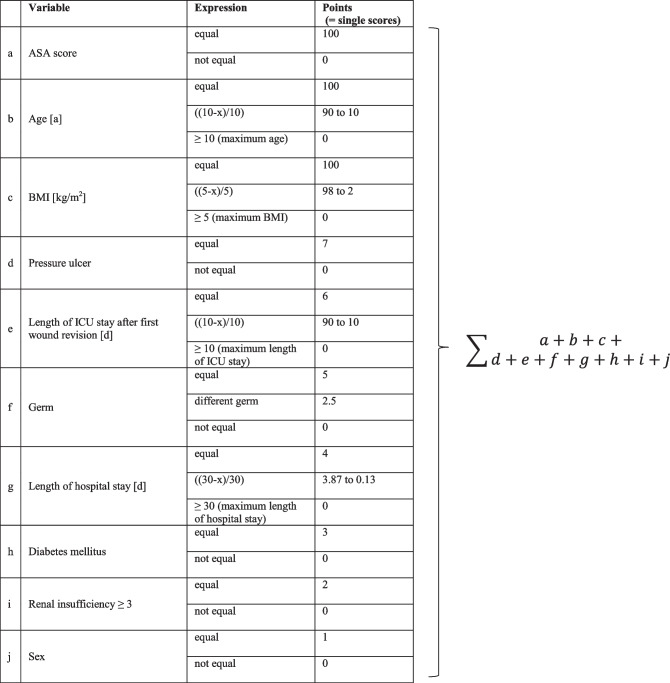
Scoring system performed for each potential match

Explanation: calculation of a single score using age:

The specified maximum age difference (10) − age difference of an epi-VAC patient and potential matching partner (*x*): maximum age difference (10)

Example: If the age difference of the matching partners is *x* = 4, the result is ((10 − 4)/10) = 60 out of 100 possible points

We used the R programming language (version 1.3.1073, © 2009-2020 RStudio, PBC) for the inferential statistical analysis. The significance level was set at *P* < 0.05.

We treated patient assignments from the matched-pair comparison analysis as linked samples. The Welch *t*-test (unrelated groups) was used for parametric continuous outcome measures or paired *t*-test (related groups). We compared two categorical variables using Fisher’s exact *t*-test (unpaired) and McNemar’s test (paired). For non-parametric and ordinal scaled characteristics, we used the Wilcoxon rank-sum test (Mann-Whitney *U* test) for the unrelated groups and the Wilcoxon signed-rank test for the matched partners [15].

## Results

A total of 218 patients were included. Of the 218 patients, 143 (65.6%) were male, and 75 (34.4%) were female. Age was (mean ± standard deviation) 65.1 ± 15.3 years, and body mass index was 28.2 ± 6.32 kg/m^2^.

Due to unequal study periods, 170 patients (78%) received conventional wound dressing for the first wound revision (2011–2016) compared with 48 (22%) patients with the epi-VAC therapy (2017–2019).

**Fig. 2:**
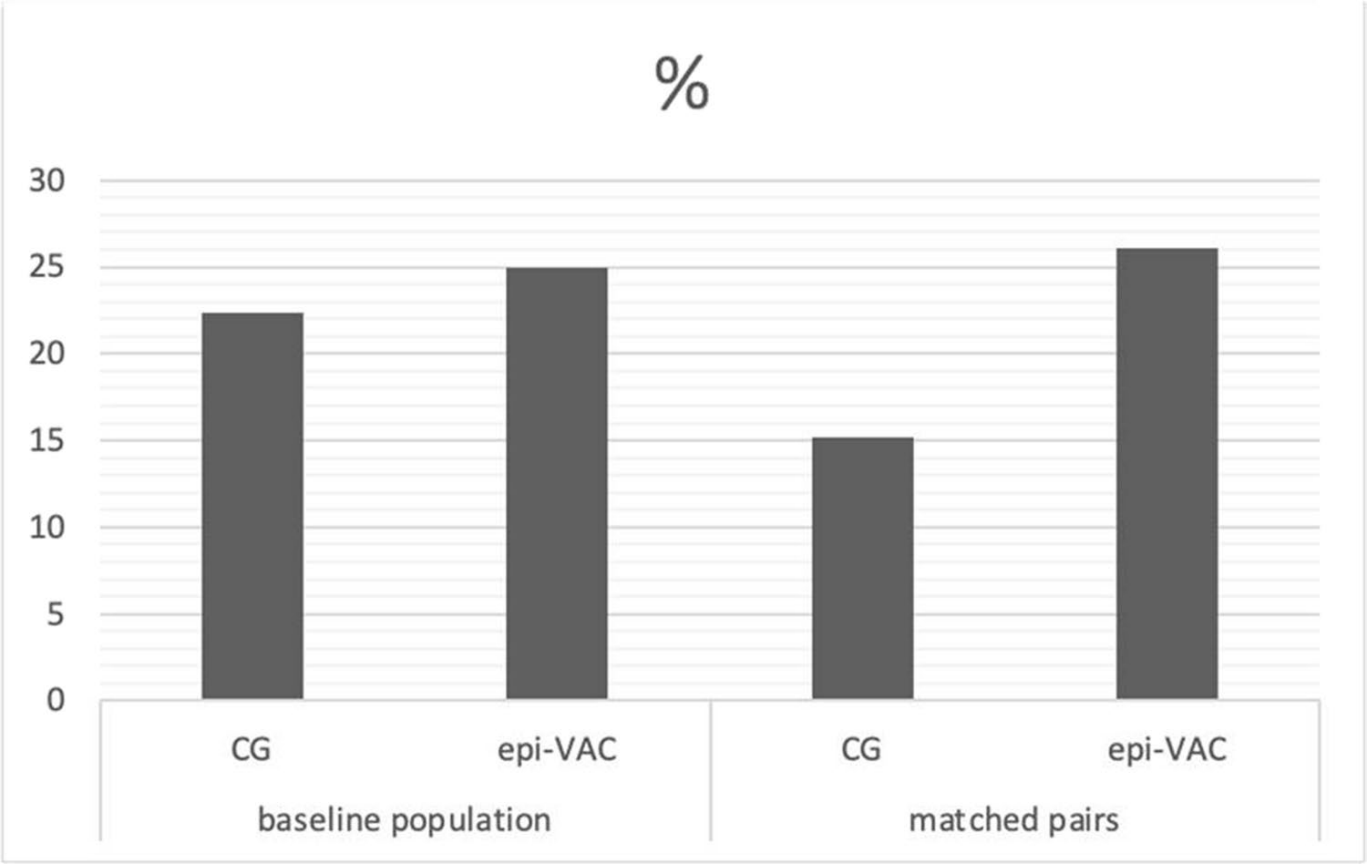
The relative proportion of treatment failures (%) in the baseline population and matched pairs compared between the control group (CG) and epi-VAC group (epi-VAC)

The use of the epi-VAC therapy after revision spine surgery did not reduce the number of further wound revisions. There was no statistically significant difference in the incidence of arbitrary revisions between the two groups (epi-VAC 12/48 (25%) to CG 38/170 (22.4%), *P* = 0.7). In the matched-pair analysis, the relative frequency nearly doubled at 26.1% in the epi-VAC group compared to that in CG at 15.2% (*P* = 0.3) (Fig. 2). However, there were no complications related to wound dressing for the epi-VAC therapy.

Table 2 (baseline population) and Table 3 (matched pairs) contain the statistical analysis of the demographic data and comorbidities.

**Table 2:** Comparison of patient- and hospital-related data between the entire collective, the epi-VAC, and the control groups

Variable	Total (*n* = 218)	Epi-VAC (*n* = 48)	Control group (*n* = 170)	*P* value
Mean age (years)	65.1 ± 15.3	68.2 ± 13.3	64.3 ± 15.8	0.08
Body mass index (kg/m^2^)	28.2 ± 6.32	29.4 ± 6.7	27.9 ± 6.2	0.16
Male (%)	65.6	54.2	68.8	0.08
ASA score	2.66	2.73	2.64	0.42
Diabetes mellitus (%)	32.6	43.8	29.4	0.08
Insulin-dependent (%)	13.8	20.8	11.8	0.15
Oral antidiabetics (%)	11.9	10.4	12.4	0.8
Pressure ulcer (%)	8.7	14.6	7.1	0.14
Nicotine abuse (%)	22	22.9	21.8	0.85
Systemic cortisone therapy (%)	9.6	10.4	9.4	0.79
Peripheral arterial disease (PAVD) (%)	4.6	2.1	5.3	0.7
Coronary artery disease (CAD) (%)	8.3	8.3	8.2	0.8
Arterial hypertension (%)	71.1	81.3	68.2	0.1
Active cancer (%)	28.9	25	30	0.59
Osteoporosis (%)	19.7	18.2	25	0.31
Renal insufficiency ≥ 3 (%)	21.6	27.1	20	0.32
Anticoagulation (%)	30.7	41.7	27.6	0.08
Albumin (g/l)	34.35 ± 7.37	31.99 ± 8.69	35.09 ± 6.77	0.08
Hemoglobin (mmol/l)	6.23 ± 1.22	5.87 ± 1.07	6.34 ± 1.25	**0.03**
C-reactive protein (mg/l)	92.41 ± 99.97	110.6 ± 98.74	87.27 ± 100	0.15
Leukocytes (× 10^9^/l)	10.03 ± 5.09	10.48 ± 6.28	9.9 ± 4.72	0.59
Wound revision (%)	22.9	25	22.4	0.7
Number of addressed vertebral bodies (*n*)	5.43 ± 2.53	5.6 ± 2.65	5.38 ± 2.5	0.6
Duration of initial surgery (min)	176.3 ± 79.95	179 ± 101.45	175.5 ± 72.8	0.83
Duration of revision surgery (min)	70 ± 32.4	71.7 ± 28.03	69.5 ± 33.59	0.65
Time between first and second WR (days)	13.6 ± 10.87	12.3 ± 7.88	14 ± 11.76	0.56
Length of hospital stay (days)	31.7 ± 21.02	39.6 ± 28.52	29.5 ± 17.84	**0.02**
ICU admission (%)	39.4	52.1	35.9	**0.04**
Local antibiotic therapy (%)	83.5	97.9	79.4	**0.01**
Germ (%)	61.5	75	57.6	**0.03**
*S*. *epidermidis* (%)	28	39.6	24.7	0.05
*S*. *aureus* (%)	21.1	27.1	19.4	0.32
Propionibacterium acnes (%)	6.9	8.3	6.5	0.75
*E*. *faecalis* (%)	6	5.3	8.3	0.49
*E*. *coli* (%)	2.8	8.3	1.2	**0.02**
*Pseudomonas aeruginosa* (%)	2.3	6.3	1.2	0.07
Another germ (%)	12.8	12.5	12.9	1
Mixed infections (%)	16.5	27.1	13.5	**0.04**
Mortality (%)	13.3	22.9	10.6	0.05

Data expressed as mean ± standard deviation or proportion in %. Significance level *P* < 0.05. Significant *P* value printed in bold

*WR* wound revision

**Table 3:** Comparison of patient- and hospital-related data of all matched patients, the epi-VAC group, and the control group after matching

Variable	Total (*n* = 92)	Epi-VAC (*n* = 46)	Control group (*n* = 46)	*P* value
Mean age (years)	68 ± 13.39	68.1 ± 13.5	67.9 ± 13.42	0.98
Body mass index (kg/m^2^)	29.27 ± 6.16	29.1 ± 6.12	29.43 ± 6.26	0.9
Male (%)	60.9	54.3	67.4	0.18
ASA score	2.7	2.7	2.7	1
Diabetes mellitus (%)	37	41.3	32.6	0.42
Insulin-dependent (%)	20.7	21.7	19.6	1
Oral antidiabetics (%)	12	8.7	15.2	0.39
Pressure ulcer (%)	8.7	13	4.3	0.13
Nicotine abuse (%)	20.7	21.7	19.6	1
Systemic cortisone therapy (%)	12	8.7	15.2	0.55
Peripheral arterial disease (PAVD) (%)	2.2	2.2	2.2	1
Coronary artery disease (CAD) (%)	14.1	13	15.2	1
Arterial hypertension (%)	82.6	84.8	80.4	0.72
Active cancer (%)	26.1	23.9	28.3	0.82
Osteoporosis (%)	22.8	26.1	19.6	0.58
Renal insufficiency ≥ 3 (%)	26.1	28.3	23.9	0.82
Anticoagulation (%)	40.2	39.1	41.3	1
Albumin (g/l)	33.02 ± 7.6	31.92 ± 8.85	34.26 ± 5.75	0.61
Hemoglobin (mmol/l)	6.05 ± 1.09	5.88 ± 1.09	6.22 ± 1.08	0.17
C-reactive protein (mg/l)	91.9 ± 88.85	114.33 ± 99.2	69.47 ± 71.38	**0.02**
Leukocytes (× 10^9^/l)	10.7 ± 5.87	10.59 ± 6.39	10.81 ± 5.38	0.78
Wound revision (%)	20.7	26.1	15.2	0.30
Number of addressed vertebral bodies (*n*)	5.4 ± 2.58	5.7 ± 2.67	5.2 ± 2.49	0.36
Duration of initial surgery (min)	177.5 ± 85.13	182 ± 102.5	172.7 ± 62.29	0.63
Duration of revision surgery (min)	67.6 ± 25.47	72.9 ± 28.01	62.3 ± 21.69	0.06
Time between first and second WR (days)	12 ± 6.92	12.3 ± 7.88	11.3 ± 5.15	0.73
Length of hospital stay (days)	32.2 ± 22.6	38.1 ± 26.03	26.2 ± 16.83	**0.01**
ICU admission (%)	42.4	50	34.8	0.15
Local antibiotic therapy (%)	88	97.8	78.3	**0.01**
Germ (%)	64.1	73.9	54.3	0.07
*S*. *epidermidis* (%)	32.6	39.1	26.1	0.27
*S*. *aureus* (%)	27.2	28.3	26.1	1
*Propionibacterium acnes* (%)	4.3	6.5	2.2	0.62
*E*. *faecalis* (%)	6.5	8.7	4.3	0.68
*E*. *coli* (%)	4.3	8.7	0	0.12
*Pseudomonas aeruginosa* (%)	2.2	4.3	0	0.49
Another germ (%)	13	13	13	1
Mixed infections (%)	21.7	26.1	17.4	0.45
Mortality (%)	16.3	23.9	8.7	0.12

Data expressed as mean ± standard deviation or proportion in %. Significance level *P* < 0.05. Significant *P* value printed in bold

*WR* wound revision

Regarding hospital-related data, we found an increased rate of patients admitted to ICU (epi-VAC 52% to CG 35.9%, *P* = 0.04) in the baseline population. The duration of hospital treatment differed significantly among the people for the respective wound dressings (epi-VAC 39.6 ± 28.5 days to CG 29.5 ± 17.8 days, *P* = 0.02). There was also a significant difference in hospitalization duration among matched patients: epi-VAC 38.1 ± 26 days to CG 26.2 ± 16.8 days, *P* = 0.01.

Otherwise, no significant statistical correlations were detectable for the duration of initial surgery, duration of wound revision surgery, number of instrumented vertebral bodies, the time between first and second wound revisions, and mortality.

In the baseline population, the proportion of patients with positive germ detection in the intra-operative wound swab (epi-VAC 75% to CG 57.6%, *P* = 0.03) was significantly higher in the epi-VAC group.

The pathogens mainly isolated in the total collective were *Staphylococcus epidermidis* (total 28% (61/218); epi-VAC 39.6%; CG 24.7%) and *Staphylococcus aureus* (total 21.1% (46/218); epi-VAC 27.1%; CG 19.4%) across groups. Mixed infections from at least two germs were present in 16.5% of cases (36/218).

In the matched-pair comparative analysis, the groups differed significantly concerning the use of a local antibiotic therapy (epi-VAC 97.8% to CG 78.3%, *P* = 0.01) (Table 3).

The values of laboratory parameters immediately before the first wound revision are also listed in Tables 2 and 3. Compared with patients in the CG, albumin levels were not significantly reduced in patients with epi-VAC dressing (epi-VAC 32 ± 8.7 g/l to CG 35.1 ± 6.8 g/l, *P* = 0.08 (total collective) and epi-VAC 31.9 ± 8.9 g/l to CG 34.3 ± 5.8 g/l, *P* = 0.61 (matching)). For the population, the Hb level in the epi-VAC group was significantly lower than that in the control group (epi-VAC 5.9 ± 1.1 mmol/l to CG 6.3 ± 1.3 mmol/l, *P* = 0.03). In the matched-pair analysis, CRP levels differed significantly between both groups (epi-VAC 114.3 ± 99.2 mg/l to CG 69.5 ± 71.4 mg/l, *P* = 0.02).

Across groups, an elevated CRP level immediately before the first wound revision was a risk factor for unplanned re-revision (135.2 ± 128.6 to 79.7 ± 86.1 mg/l, *P* = 0.0057). The demographic data and comorbidities of all patients suffering an unplanned re-revision surgery are shown in  Table 4.

**Table 4:** Comparison of patient- and hospital-related data of all matched patients with a new unplanned wound revision depending on the wound dressing

Variable	Treatment failures		
Wound dressing	Total (*n* = 19)	Epi-VAC (*n* = 12)	Control group (*n* = 7)
Mean age (years)	65.7 ± 13.6	67.2 ± 10.2	63.1 ± 18.7
Body mass index (kg/m^2^)	30.4 ± 6.9	32.36 ± 6.7	26.93 ± 6.1
Male (%)	63.2	50	85.7
ASA score	2.7	2.7	2.7
Diabetes mellitus (%)	47.4	58.3	28.6
Insulin-dependent (%)	36.8	50	14.3
Oral antidiabetics (%)	0	0	0
Pressure ulcer (%)	15.8	25	0
At least one concomitant disease (%)	89.5	100	71.4
Nicotine abuse (%)	21.1	16.7	28.6
Systemic cortisone therapy (%)	5.3	0	14.3
Peripheral arterial disease (PAVD) (%)	5.3	0	14.3
Coronary artery disease (CAD) (%)	15.8	16.7	14.3
Arterial hypertension (%)	84.2	91.7	71.4
COPD (%)	10.5	0	28.6
Active cancer (%)	26.3	16.7	42.9
Osteoporosis (%)	21.1	25	14.3
Renal insufficiency ≥ 3 (%)	31.6	33.3	28.6
Anticoagulation (%)	5.3	25	14.3
Albumin (g/l)	28.8 ± 7.23	28.47 ± 7.77	29.45 ± 6.63
Hemoglobin (mmol/l)	5.79 ± 1.05	5.76 ± 1.03	5.84 ± 1.16
C-reactive protein (mg/l)	161.94 ± 117.18	186.66 ± 128.92	119.56 ± 86.04
Leukocytes (× 10^9^/l)	10.95 ± 3.45	10.9 ± 4.12	11.04 ± 2.13
Number of addressed vertebral bodies (*n*)	5.6 ± 3.1	5.2 ± 2.7	6.4 ± 3.8
Duration of initial surgery (min)	169.6 ± 65.2	152.8 ± 55.5	203.2 ± 75
Duration of revision surgery (min)	63.6 ± 16.1	60.4 ± 15.8	69 ± 16.5
Time between first and second WR (days)	11.7 ± 7	12 ± 8.1	11.3 ± 5.2
Length of hospital stay (days)	49.9 ± 29.2	51 ± 31.6	48 ± 26.9
ICU admission (%)	47.4	50	42.9
Length of ICU stay (days)	8.8 ± 17.8	11.5 ± 21.9	3.3 ± 1.2
Mortality (%)	10.5	8.3	14.3
Local antibiotic therapy (%)	100	100	100
Germ (%)	78.9	75	85.7
*S*. *epidermidis* (%)	21.1	16.7	28.6
*S*. *aureus* (%)	42.1	33.3	57.1
*Propionibacterium acnes* (%)	0	0	0
*E*. *faecalis* (%)	10.5	16.7	0
*E*. *coli* (%)	10.5	16.7	0
*Pseudomonas aeruginosa* (%)	0	0	0
Another germ (%)	26.3	25	28.6
Mixed infections (%)	26.3	25	28.6

Data indicated as mean or percentage

*PAVD* peripheral arterial disease, *COPD* chronic obstructive pulmonary disease, *WR* wound revision

## Discussion

We are unaware of any current study addressing the benefit of the epi-VAC therapy after spinal wound revision. Our retrospective study found no effect of the epi-VAC therapy on the rate of unscheduled re-revision surgery.

We compared the epi-VAC group with a CG whose patients received a standard wound dressing. There was no statistically significant difference between the groups regarding the proportion of treatment failures in the one-to-one matched-pair comparison analysis.

We considered a variety of characteristics commonly cited in the literature as risk factors for wound-healing disorders. One exogenous factor that has a compromising effect on wound healing is evidence of bacterial colonization in the wound swab [16]. We expected the epi-VAC to seal the wound and reduce bacterial colonization effectively [17]. This could lead to improved wound healing and a lower rate of wound-related revision surgery. However, in our work, bacterial colonization was statistically significantly different between the epi-VAC and the CG in the overall population (epi-VAC 75% to CG 57.6%, *P* = 0.03).

Other key proved active principles are the absorption of excess wound exudate [18], a better adaptation of the wound edges [19], and improvement of microcirculation [20]. However, according to the studies available in the literature, no significant reduction in the rate of re-operation could be observed after applying epi-VAC on spinal wounds despite these properties [6, 7].

That leads to the question of why the previously published favourable properties of the epi-VAC dressing on wound healing are not reflected in the spine revision and re-revision rate.

An elevated CRP value was a risk factor for repeat wound revision surgery across groups in our study. That infection in the surgical area after dorsal spondylosyndesis shows typical kinetics of post-operative CRP progression was described by Hoeller et al. [21]. A severely elevated CRP level or reduced decline indicates fulminant infections. We suspect that a single wound revision without implant exchange might not be sufficient in such cases, and a follower-revision surgery would become necessary. In our study, the implants were regularly changed if another revision surgery was required (re-revision).

The advantage of the epi-VAC therapy in spinal surgery seems to reduce relatively mild wound-healing disorders. As they have a prolonged but eventually regular course, they are not worthy of surgery, as postulated in several preliminary studies [6, 7].

### Limitations

Due to the retrospective study design and the associated dependence on correct and complete data collection, we could not collect incriminating data on the benefit of the epi-VAC therapy for low-grade wound-healing disorders. Another weakness is the small number of patients receiving the epi-VAC treatment (48). In this regard, our matching method proved to be a good option concerning the established criteria to achieve the best possible comparability of the two groups.

The different durations of the epi-VAC therapy affected the results, which varied from three to ten days. In addition, the proportion of patients receiving topical antibiotic therapy differed significantly between the groups. With future research on this type of wound dressing, it would be desirable to standardize the duration of use and topical antibiotic treatment (ideally prospective multicenter). Moreover, an important focus of further studies should be the question of which groups of patients would benefit from the epi-VAC dressing in spine surgery.

## Conclusions

In summary, evaluating the total collective and matched pairs, the relative proportion of treatment failures was higher in the epi-VAC group than in the control group. Our study design could not verify the impact of the epi-VAC therapy in preventing the rate of unexpected re-revision surgeries. An elevated CRP level immediately before first revision surgery was a risk factor for further unplanned wound revision across groups.

## Data Availability

The datasets generated and analyzed during the study are available from the corresponding author on reasonable request.
